# Design and evaluation of a novel direct hemagglutination test based on a recombinant protein for diagnosis of cystic echinococcosis

**DOI:** 10.1186/s13071-025-06900-1

**Published:** 2025-07-08

**Authors:** Abolfazl Masoumi Koushk Mehdi, Hossein Motedayyen, Majid Fasihi Harandi, Hossein Akbari, Amin Moradi Hasan-Abad, Mohsen Arbabi

**Affiliations:** 1 Department of Medical Parasitology, School of Medicine, Kashan University of Medical Sciences, Kashan, Iran; 2 Autoimmune Diseases Research Center, Kashan University of Medical Sciences, Kashan, Iran; 3 Department of Public Health, School of Hygiene, Kashan University of Medical Science, Kashan, Iran; 4 Health Information Management Research Center, Kashan University of Medical Science, Kashan, Iran; 5 Research Center for Hydatid Disease in Iran, Afzalipour School of Medicine, Kerman University of Medical Sciences, Kerman, 76169-14111 Iran

**Keywords:** Cystic echinococcosis, Human, Recombinant protein, Hemagglutination, Diagnosis, Direct

## Abstract

**Background:**

Cystic echinococcosis (CE), one of the most important parasitic diseases, threatens global health for animals and, occasionally, humans. Accurate and timely diagnosis is crucial for effective disease management and control. Therefore, this study focused on designing and investigating a novel direct hemagglutination test (HAT) using a recombinant protein to diagnose hydatid disease.

**Methods:**

The fusion protein IH4_Antigen_HisTag was designed by incorporating EgFABP1, EgTeg, and IH4 nanobody sequences. The fragment was cloned for expression in *Escherichia coli * BL21 (DE3) using the vector pET-28a. It was optimized through isopropyl β-d-thiogalactopyranoside (IPTG) induction. The results of a transfection were assessed using sodium dodecyl sulfate polyacrylamide gel electrophoresis (SDS-PAGE) and western blotting. For consistency, a HAT was developed using O-negative red blood cells (RBCs) with standardized antigen concentrations. The sensitivity and specificity of the test were assessed by the receiver operating characteristic (ROC) curve, using serum samples from 43 patients with CE and 43 healthy controls.

**Results:**

The results of SDS-PAGE and western blot revealed that the size and concentration of the produced protein were 27 KDa and 126.1 μg/ml, respectively. The highest sensitivity (95%) and maximum specificity (95.3%) were observed at the cutoff point of the HAT equal to 0.09375 [area under the curve (AUC) = 0.972].

**Conclusions:**

The HAT, as a test with low cost, ease of use, and no need for advanced equipment, has acceptable sensitivity and specificity in determining antibodies against the parasite. The IH4-Antigen-HisTag protein can be used in healthcare systems as a simple, cost-effective diagnostic tool for hydatid disease.

**Graphical Abstract:**

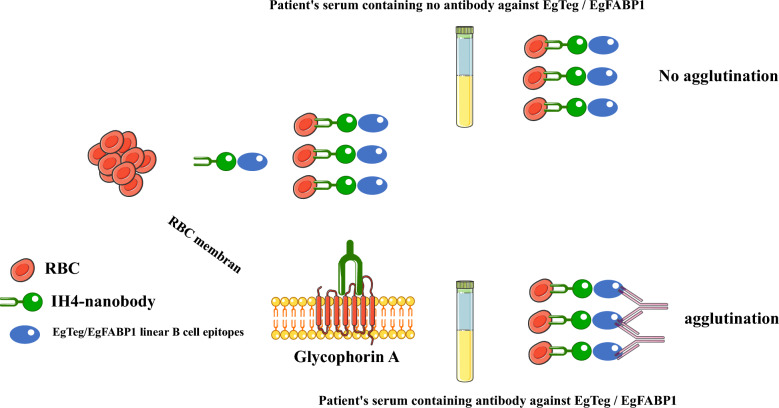

**Supplementary Information:**

The online version contains supplementary material available at 10.1186/s13071-025-06900-1.

## Background

Infectious cystic echinococcosis (CE) occurs from the larval stage of the *Echinococcus granulosus* sensu lato. Humans are dead-end hosts, where larval cysts primarily develop as fluid-filled cysts in the liver and lung [1]. People are infected through ingesting parasite eggs in contaminated food, water, soil, or through direct contact with dogs as definitive hosts [2]. CE in humans is a chronic, clinically complex, and neglected disease [3]. A transmission pathway exists between canid definitive hosts and livestock, primarily with sheep as intermediate hosts [4]. The disease is a significant health problem and causes economic losses worldwide, such as in the Middle East [5–7]. This disease develops in various organs such as liver, lung, brain, kidney, bone, and others [8].

According to estimates, CE causes a loss of approximately 1 million disability-adjusted life years (DALYs). However, accurately determining the prevalence and number of infected individuals, essential for disease burden calculations, remains challenging. The execution of comprehensive, population-wide studies on CE is partly hindered by the complex socio-epidemiological characteristics of the infection and the insufficiency of current diagnostic tools for effectively mapping disease distribution at the population level [5, 9, 10].

The diagnosis depends on the clinical findings, imaging (radiology, ultrasonography, computed axial tomography, and magnetic resonance imaging), and serology [11]. Ultrasound is the primary imaging tool for detecting abdominal cysts; however, the limited availability of equipment and trained specialists, such as radiologists, often hinders accurate diagnosis, and other imaging techniques have limitations [12]. Serological tests can be used when imaging findings are inconclusive [13, 14]. Owing to the difficulty of managing CE using imaging technology, serological tests are a complement. Some studies demonstrated that fusion recombinant antigens could enhance serodiagnosis and be helpful in managing CE and its pulmonary form [15–18].

Antibodies (Ab) against CE can be detected using enzyme-linked immunosorbent assays (ELISAs) and indirect hemagglutination (IHA) [19], which can provide enough sensitivity. However, false positive results are a challenge, perhaps due to the presence of antibodies from previous infections or cross-reactivity with other parasitic diseases [20]. In contrast, antigen (Ag) detection methods provide higher specificity and better correlation with active infection conditions, making them particularly valuable for monitoring disease progression and treatment efficacy [21]. However, Ag detection tests may have lower sensitivity, especially in the early stages of infection and in case of low parasite burden [20]. Therefore, choosing Ab and Ag detection approaches depends on the clinical context and diagnostic needs.

The increasing demand for rapid, affordable, and user-friendly diagnostic tools for diagnosing CE proposes the need for developing innovative approaches, particularly in endemic regions with weak laboratory infrastructure [22, 23]. This request is especially critical in some countries such as Iran, where the seroprevalence of human CE is estimated at approximately 4%, with substantial heterogeneity across regions and populations [24].

Hemagglutination-based assays (HATs) and other rapid diagnostic tests (RDTs) are emerging as practical solutions for point-of-care diagnosis, field screening, and early detection of infections [25]. Despite the promising features of RDTs, challenges remain regarding standardization and variability in diagnostic performance based on cyst stage and anatomical location [26]. Addressing these issues is crucial to optimizing rapid tests’ utility across diverse clinical and field settings. Among serological methods, the HAT presents notable advantages for large-scale epidemiological surveys, including low cost, ease of use, and minimal equipment requirements. It is particularly suitable for application in resource-limited areas [27].

HAT-based assays have been successfully utilized in parasitology to recognize antibodies against pathogens in Chagas disease and human malaria [28, 29]. Few studies have investigated HATs in CE, providing a unique opportunity to develop a new diagnostic method [19, 30, 31]. Although this method may not have the highest sensitivity, its notable specificity and practical advantages suggest the potential for further refinement as complementary tools to existing diagnostics [19, 32].

Various antigens have been used for CE diagnosis, including antigen B (AgB), antigen 5, and hydatid cyst fluid; however, their diagnostic efficacy needs more investigation [18, 33]. Cross-reactivity between the antigens of *E. granulosus* and those from other platyhelminths, such as *E. multilocularis*, *Taenia solium*, and *Fasciola hepatica*, reduces the specificity of serological assays for CE. Consequently, recombinant antigen-based diagnostic approaches are being pursued. Researchers have designed and evaluated various recombinant antigens from this parasite [16, 17, 34]. The use of fusion recombinant proteins, particularly when it consists of multiple specific antigens, can develop the efficiency of serodiagnosis tests [35]. In some areas where CE is prevalent, recombinant antigens have been suggested, as they have shown better results in diagnosing the disease than complete parasite antigens [36].

The EgTeg, a tegumental protein expressed during the larval stage of *E. granulosus*, has been identified as a promising diagnostic and vaccine candidate owing to its multiple epitopes and capacity to inhibit neutrophil chemotaxis, thereby facilitating parasite survival [37, 38]. It is expressed on both the protoscolex and the cyst wall. Also, EgFABP1, a protein expressed throughout the parasite’s life cycle, plays a vital role in fatty acid uptake and stimulates immunoglobulin E (IgE) production, making it another strong candidate for serodiagnosis and vaccine development [38, 39].

Previous studies have suggested that a combination of several antigens can be helpful to improve the sensitivity and specificity of serological tests [15–18]. A prior study focusing on vaccination with recombinant antigens composed of EgTeg and EgFABP1 proteins have shown that these proteins can induce the production of both IgE and IgG4 antibodies [38]. Accordingly, the present study focused on expressing a recombinant fusion protein containing eight B cell linear epitopes from the EgFABP1 and EgTeg proteins fused to IH4 (IH4 V_H_H, or nanobody, recognizes human glycophorin A [40]) expressed in *E. coli* BL21 (DE3) to develop and evaluate a hemagglutination assay.

## Methods

### Epitope selection and recombinant protein design and gene synthesis

The design and construction of the IH4_Antigen_HisTag fusion protein was guided by comprehensive bioinformatics analyses. The amino acid sequence and the plasmid map are presented in the supplementary materials (Supplementary Fig. S1 and Supplementary Table S1). Briefly, the gene sequences of EgFABP1, EgTeg, and IH4 nanobody were obtained from the GenBank database. These proteins were analyzed using the Immune Epitope Database (IEDB) software to identify B lymphocyte epitopes. Subsequently, fusion proteins were designed, and their corresponding genes were cloned into the pET-28a expression vector using XhoI and NcoI restriction sites. The final gene construct, encoding the IH4_Antigen_HisTag fusion protein, was synthesized in collaboration the Pishgam Company (Tehran, Iran). The plasmid map of this construct is provided in Supplementary Fig. S1.

### Transformation of susceptible BL21 (DE3) bacteria with pET-28a vectors containing the recombinant protein gene sequence

The recombinant protein was expressed using *E. coli* BL21 (DE3). Plasmid transformation was performed using the calcium chloride/heat shock method, and transformed colonies were selected on Luria Bertani (LB) agar supplemented with 50 µg/ml kanamycin. Following transformation, plasmid DNA was extracted from selected colonies using a plasmid extraction kit (DNAbiotech, Iran). The extracted plasmids were subsequently analyzed by agarose gel electrophoresis to confirm their presence and size (Supplementary Fig. S2). To ensure the reliability of our results, BL21 (DE3) cells without plasmid were used as a negative control.

### Expression of recombinant IH4_Antigen_ HisTag

The colonies of plasmid-carrying bacteria were cultured in LB broth supplemented with 50 μg/ml kanamycin at 37 °C for 24 h. To optimize the expression conditions of the recombinant protein, a wide range of conditions were tested. Bacterial cultures at optical densities (OD600) ranging from 0.1 to 0.6 were induced with different concentrations of IPTG (0.5 to 8 mM) and incubated for 5, 10, 15, and 20  h. The bacteria biomass was harvested by centrifugation and resuspended in phosphate-buffered saline (PBS). The mixture was then incubated at −80 °C for 10 min and thawed at 4 °C. The mixture was frozen and thawed three more times to facilitate the cell wall decomposition and then sonicated on ice at ultrasonic level 6.0, 10 × 30 s, followed by centrifugation at 12,000 *g*, 20 min. The collected supernatant was stored at −20 °C. Protein expression levels were evaluated using SDS–polyacrylamide gel electrophoresis (SDS-PAGE) and western blot analysis.

### SDS-PAGE and western blotting of the recombinant protein

Protein samples (40 μl) were mixed with 10 μL of 5× loading buffer, boiled for 5 min, and subsequently loaded onto a 12% SDS-PAGE gel. Electrophoresis was initially conducted at 60 V for approximately 62 min until samples migrated into the resolving gel, followed by an increased voltage of 120 V until the crucial step of complete separation of the protein marker bands was achieved. Afterward, the gel was stained with Coomassie Brilliant Blue R-250 for 2 h and destained overnight.

For western blot analysis, proteins were transferred onto a PVDF membrane using a semi-dry transfer system at 25–30 V for 2 h. The membrane was then blocked with 5% skimmed milk in PBST (PBS containing 0.05% Tween-20) for 1 h at room temperature. The His-tagged recombinant protein was detected using an anti-HisTag-HRP conjugated antibody at a 1:2000 dilution for 1 h. Then, the blot was developed using DAB, which generated a brown-colored product. The blot was placed in a fresh container and covered with H_2_O_2_/DAB solution for 10–15 min. The reactions were stopped by rinsing in dH_2_O while wet, then the PVDF membrane was dried at room temperature.

### Protein measurement by the Bradford method

Protein concentrations were determined using the Bradford assay, which was performed according to the original protocol [41, 42]. This is a colorimetric method based on Coomassie Brilliant Blue R-250 dye binding to proteins. Absorbance was measured at 595 nm, and protein concentrations were calculated using bovine serum albumin (BSA) as a standard.

### Hemagglutination kit development

#### Cell source of red blood cells

Whole blood, anticoagulated with EDTA, from the O-negative blood group was used as the source of red blood cells (RBCs). Human O-negative blood was selected owing to the absence of A, B [43], and Rh antigens [44], which minimizes the risk of nonspecific agglutination reactions. This selection enhances the specificity of HAT by reducing background interference and ensuring that agglutination results primarily from specific antigen–antibody interactions [45]. Initially, whole blood was centrifuged at 2000 rpm for 10 min at 4 °C using a refrigerated centrifuge to maintain RBC integrity [46]. The plasma and buffy coat were then carefully removed. The remaining RBCs were washed by adding an equal volume of PBS, followed by centrifugation, and the supernatant was discarded. This washing step was repeated two to three times [47]. Finally, the washed RBCs were diluted in isotonic PBS to prepare two suspensions at dilutions of 1:40 and 1:80, the latter being approximately twice as concentrated.

#### Sensitivity and specificity testing

Our assessment of the sensitivity and specificity of the HAT was conducted in collaboration with the Biobank of the Hydatid Cyst Research Center at Kerman University of Medical Sciences and the Department of Surgery at Shahid Beheshti Hospital, Kashan University of Medical Sciences. We obtained 43 positive plasma samples from individuals diagnosed with CE via imaging techniques from these reputable institutions. In addition, we collected 43 negative control samples according to the manufacturer’s instructions, from individuals confirmed to be seronegative for CE using a commercial ELISA kit (Pishtaz Teb Diagnostics, Iran).

A total of 86 samples were re-numbered and blinded using the www.kitset.ir random number sampling tool to eliminate bias during results interpretation. Following the reassignment, a worklist was generated on the basis of the new identifiers. The HAT was performed on V-shaped plates, with 11 samples and 1 negative control per plate. The concentration of antigen and volume of the selected RBC suspension were standardized and calculated for each test batch accordingly.

**Fig. 1:**
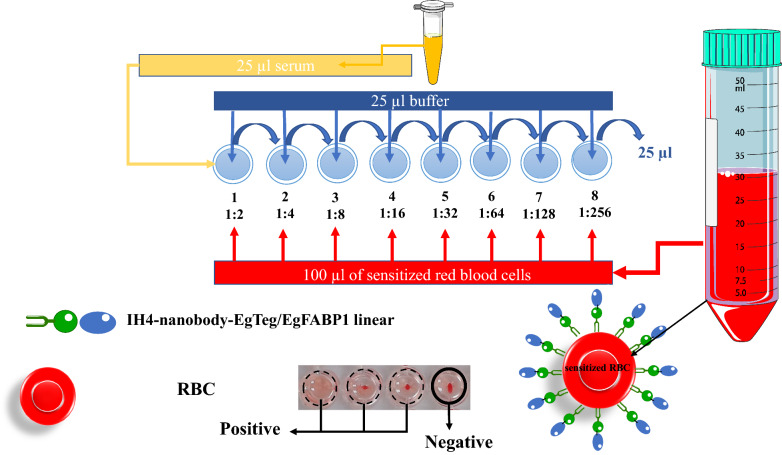
Schematic image of sera dilutions and kit protocol

Samples were diluted (Fig. 1) using a negative control serum obtained from an individual without paraclinical evidence (e.g., computed tomography [CT] scan) and confirmed negative for CE by ELISA. For each assay, the volumes of RBCs and antigen were calculated, mixed in a 50 ml Falcon tube, and incubated on a shaker for 30 min. During this incubation phase, the recombinant antigen binds exclusively to glycophorin A found on the surface of RBCs, mediated by IH4, which sensitizes the cells [40]. Following sample dilution, 100 µl of the antigen-sensitized RBC suspension was added to each well and incubated for 1 h at 25 °C. For the final reading, the plate was tilted for 30 s to 1 min (Fig. 1).

In the HAT, a compact “button” at the bottom of the well was interpreted as a positive agglutination reaction, indicating the presence of specific antibodies. Conversely, forming a “teardrop” pattern upon tilting the plate signified the absence of agglutination and was considered a negative result.

### Statistical analysis

The SPSS software version 22 (Armonk, NY: IBM Corp) was employed to determine the optimal cutoff value and the performance of the HAT in disease diagnosis using a receiver operating characteristic (ROC) curve. The area under the ROC curve (AUC) was calculated to assess the overall diagnostic accuracy of the test. The optimal cutoff point was identified on the basis of the Youden index, maximizing the balance between sensitivity and specificity. Subsequently, a confusion matrix was constructed to calculate the sensitivity and specificity of the test at the selected cutoff value.

## Results

### Cloning and transformation of *E. coli* BL21 (DE3) with pET-28a vectors containing recombinant protein sequences

The bacterial transformation assay using *E. coli* BL21(DE3) and the pET-28a plasmid was evaluated by selecting and screening colonies on LB agar plates supplemented with 50 μg/ml kanamycin. The presence of numerous single colonies on kanamycin-containing plates indicated successful transformation and expression of the kanamycin resistance gene. Appropriate controls confirmed the validity of the assay: no growth occurred on kanamycin plates inoculated with non-transformed cells (negative control), while growth was observed on antibiotic-free plates inoculated with non-transformed cells (positive control). In total, 19 colonies carrying the pET-28a plasmid were successfully isolated on kanamycin-containing media. Representative images of these transformed colonies are provided in the Supplementary Materials (Supplementary Figs. S3 and S4).

### Investigation and confirmation of recombinant protein gene expression with SDS-PAGE and western-Blot

The electrophoresis results demonstrated the expression of the desired recombinant protein. A protein band size of approximately 33 kDa in IPTG-induced samples, which was not present in the negative control, confirmed this finding.

**Fig. 2:**
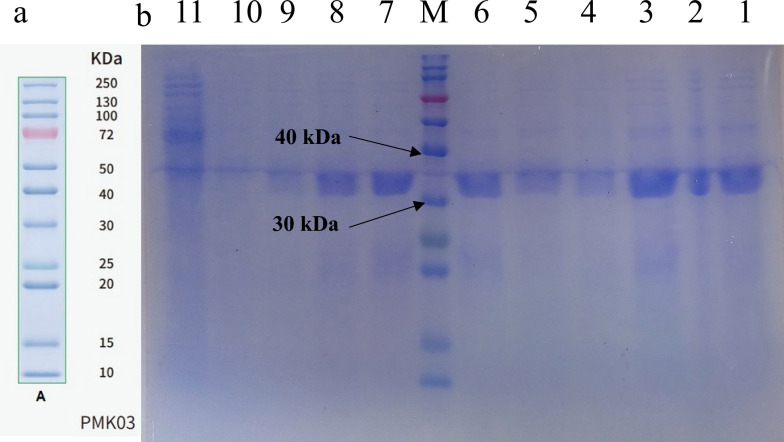
Results of the SDS-PAGE of transformed and gene-expressing colonies with IPTG. **a** Protein marker (size of protein marker bands with code PMK03). **b** SDS-PAGE gel image [SDS-PAGE analysis of recombinant protein expression. Lanes 1–9: *E. coli* BL21 (DE3) colonies transformed with pET-28a IH4_Antigen_HisTag plasmid and induced with IPTG; lane 10: uninduced plasmid-containing control; lane 11: negative control (non-transformed *E. coli* BL21(DE3)]

For initial screening purposes, recombinant protein expression was evaluated directly from crude bacterial lysates without purification. Efforts focused on optimizing expression conditions and minimizing impurities through sterile techniques. In the SDS-PAGE gel (Fig. 2), lanes 1 to 9 represent colonies expressing the recombinant protein, lane 10 represents non-induced plasmid-containing bacteria, and lane 11 reveals bacteria lacking the recombinant plasmid.

**Fig. 3:**
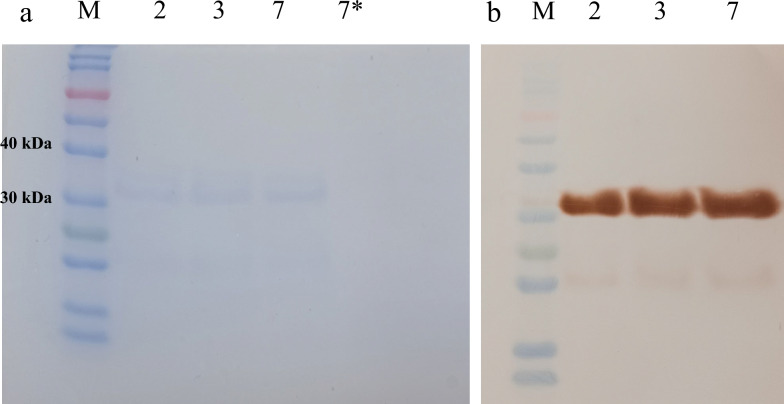
The SDS PAGE and western blot results of samples 2, 3, 7, and 7*. **a** SDS-PAGE gel image (the bands are less clear, due perhaps to the change in the ratio of the amount of protein to the loading buffer). **b** PVDF paper image (clear brown bands were further confirmation of the recombinant protein with anti-polyhistidine tail antibody). SDS-PAGE (left) and western blot (right) analysis of selected colonies. Lanes include the protein marker; induced colonies 2, 3, and 7; and an uninduced control (7*); the western blot confirmed the expression of the IH4_Antigen_HisTag protein at approximately 33 kDa in induced samples

The samples were tested in two stages. The first stage involved SDS-PAGE followed by gel staining (Fig. 3a). In the second stage, SDS-PAGE was performed under the same conditions; however, the proteins were transferred to a PVDF membrane, which was then blocked and incubated with a specific anti-polyhistidine tag antibody. Detection was performed using DAB tablets instead of TMB (Fig. 3b). A band at approximately 33 kDa was observed in the western blot, consistent with the SDS-PAGE results. This confirmed the antibody’s specific binding to the target protein and verified the presence of the recombinant protein (Fig. 3).

The analysis of colonies numbered 2, 3, and 7 is presented in Fig. 3, which showed expression bands during the initial SDS-PAGE screening (Fig. 2). These colonies were re-cultured, induced again for recombinant protein expression, and subsequently analyzed by SDS-PAGE and western blotting to confirm the presence of the IH4_Antigen_HisTag protein. The left panel of Fig. 3 shows the SDS-PAGE gel after blotting and re-staining, displaying the molecular weight marker followed by samples 2, 3, and 7. A negative control sample (shown as 7*) was included, representing bacteria carrying the plasmid without induction. The right panel of Fig. 3 shows the western blot results, where brown-colored bands at approximately 33 kDa confirmed the expression of the His-tagged recombinant protein in the induced samples.

### Bradford test results

Bradford test results, based on the obtained calibration curve, showed that the recombinant protein concentration was calculated to be 126.1 μg/ml.

### Diagnostic sensitivity, and specificity of the HAT

**Fig. 4:**
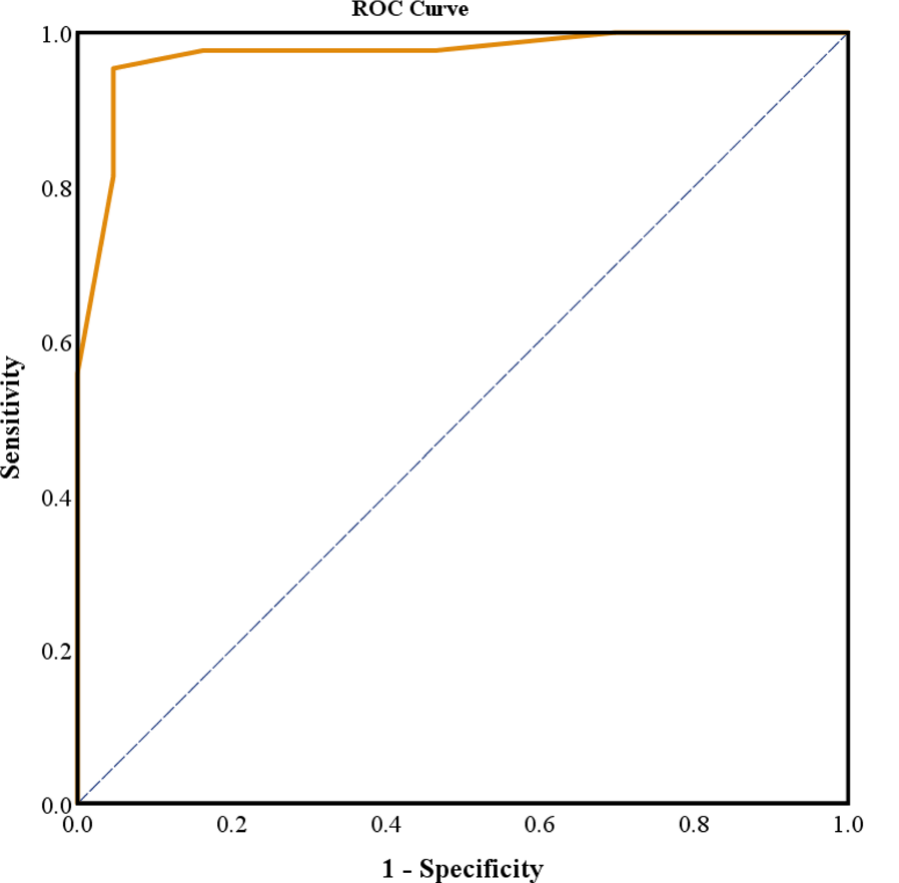
ROC curve of hemagglutination test for disease diagnosis

In this study, the performance of the HAT for CE diagnosis was evaluated by calculating its sensitivity and specificity. On the basis of the ROC analysis for the HAT (Fig. 4), the AUC was 0.972, indicating high accuracy in distinguishing between positive and negative cases. At a cutoff point of 0.09375, the test showed 95% sensitivity and 95.3% specificity, with positive and negative predictive values (PPV and NPV) of 91.1% and 95.1%, respectively. A lower cutoff of 0.04687 increased sensitivity to 97.7% but reduced specificity to 83.7%, with PPV and NPV of 94.7% and 85.4%, respectively. These results demonstrated the performance of the HAT in disease diagnosis. The findings suggest that this test can be used both qualitatively to determine positive or negative status and quantitatively to assess antibody titers for diagnosing and monitoring CE. For this purpose, serum samples are initially tested at the selected cutoff point to determine the presence or absence of CE based on the formation of a button. If positive, serial dilutions are prepared and tested to determine the highest positive antibody titer, which is then specified and reported.

As shown in the Table 1, the AUC was 0.972, indicating high accuracy in distinguishing between positive and negative samples. The standard error was 0.017, reflecting the precision of the AUC estimate. The statistical significance value (*P*-value) was 0.000, demonstrating the significance of the results. The 95% confidence interval ranged from 0.938 to 1.000, confirming the reliability of the findings. These results validated the high efficiency of the developed HAT.

**Table 1:** ROC analysis for hemagglutination test

Area under the curve				
Test result variable(s): HAT				
Area	Std. error^a^	Asymptotic sig.^b^	Asymptotic 95% confidence interval	
			Lower bound	Upper bound
0.972	0.017	0.000	0.938	1.000

The test result variable(s): HAT has at least one tie between the positive actual state group and the negative actual state group. Statistics may be biased

^a^ Under the nonparametric assumption

^b^ Null hypothesis: true area = 0.5

Table 2 indicates that lowering the cutoff point increased sensitivity but decreased specificity. Choosing an appropriate cutoff depends on whether sensitivity or specificity is prioritized. According to Youden’s index calculation, the cutoff point 0.09375 had a higher Youden’s index (0.903), indicating a better balance between sensitivity and specificity and improved test performance. This cutoff may be more suitable in cases where balancing detecting true positives and minimizing false negatives is critical.

**Table 2:** Comparison of sensitivity and specificity based on different cutoff points

Cutoff	AUC (%)	Sensitivity (%)	Specificity (%)	PPV (%)	NPV (%)
0.09375	97.2	95	95.3	91.1	95.1
0.04687		97.7	83.7	94.7	85.4

**Fig. 5:**
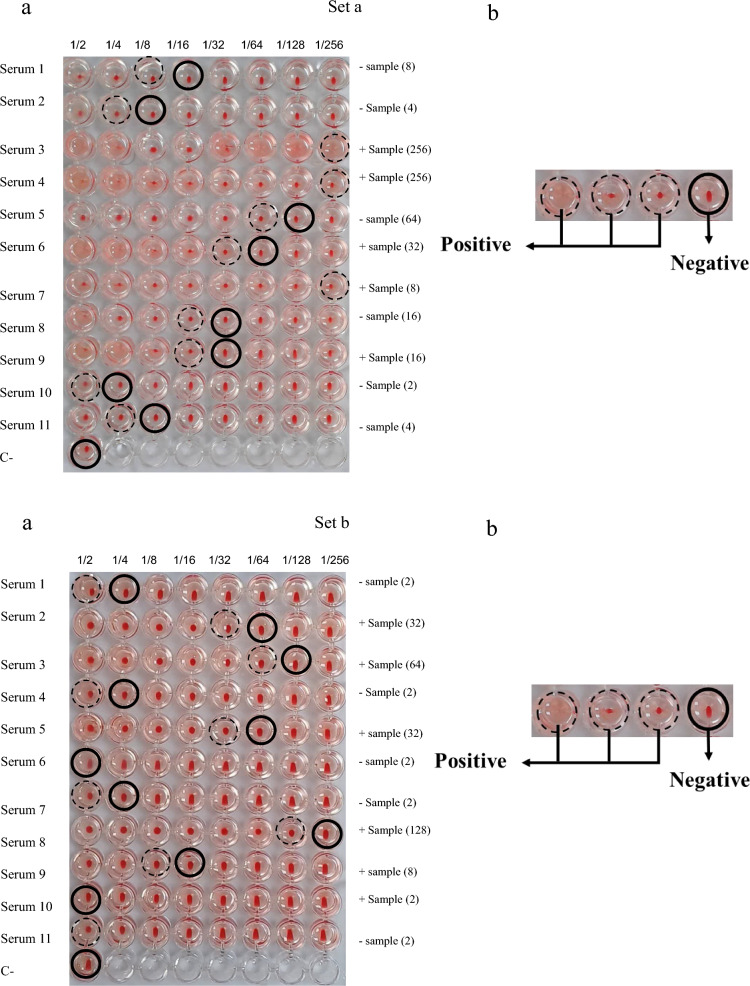
Haemagglutination test (HAT) results are displayed in two sets (a and b), each containing panels **a** and **b**. In both sets, panel **a** shows an agglutination reaction, while panel **b** shows a control result (positive and negative)

In the HAT for diagnosing CE in the analyzed samples, various results were observed (Fig. 5). The figure shows two sets of randomly selected samples under investigation. The results demonstrated that some samples exhibited a strong positive reaction, detectable even at dilutions up to 1:256, indicating a high level of antibodies against the produced recombinant antigens. Conversely, some samples showed no hemagglutination reaction even at lower dilutions, indicating a negative response. The negative control confirmed the absence of reaction, supporting the specificity of the test. These findings suggest that the HAT can be effectively used for CE identification and, on the basis of the conducted analyses, possesses favorable sensitivity and specificity.

## Discussion

CE is a parasitic infection primarily caused by *E. granulosus* and is considered a significant public health concern in many regions worldwide, including Iran [48]. The disease is asymptomatic in its early stages but can have substantial economic consequences, particularly due to veterinary treatment costs and its high prevalence among livestock [49]. Current diagnostic approaches include imaging techniques such as ultrasound, CT, and MRI, as well as serological tests such as ELISA and agglutination. Nevertheless, noninvasive ultrasonography and other imaging methods are not always available. In this context, serological tests have a complementary role, potentially confirming cyst diagnosis when there is unclear or ambiguous imaging data [50, 51].

Currently, serological assays predominantly rely on identifying antigens extracted from cyst fluid, which presents difficulties in antigen availability [52]. Consequently, developing recombinant antigens can provoke particular immune responses, enhancing the precision and efficacy of a CE serological diagnosis.

In the present study, the gene encoding this recombinant fusion protein from *E. granulosus* was expressed in *E. coli* BL21 (DE3). After protein isolation, the recombinant protein was employed as an antigen for diagnosing CE. The HAT was used to assess the antigenicity of the recombinant protein. In previous studies, recombinant multi-epitope fusion proteins have been used to detect CE antibodies, but the antigen evaluation method in those studies was an ELISA [16, 17, 53].

The results of SDS-PAGE revealed a protein band around 33 kDa, which was approximately 5 kDa larger than the molecular weight estimated by the bioinformatic software that predicted approximately 27.8 kDa. This discrepancy can be attributed to large amounts of glycine and serine residues (34.6%) and the presence of a polyhistidine tag in the recombinant fusion protein, which is unable to bind SDS, thereby affecting the size and mobility of the protein on the gel [54, 55]. Furthermore, the presence of the polyhistidine tag in the protein was confirmed by western blot analysis.

A significant factor in diagnosing CE is the development of cost-effective serological tests to detect immune responses. Many advanced diagnostic methods for CE are often inaccessible in resource-limited settings. Despite their initial promise for rapid and simple diagnosis, some existing approaches, such as lateral flow assays, frequently exhibit suboptimal performance, high costs, and variability between batches. In contrast, the HAT presents several advantages [56]. The cost-efficiency and simplicity of the HAT are significant advantages in diagnosing CE, positioning it as a suitable screening method for use in resource-limited environments [57].

Our research indicated that this technique successfully recognized antibodies in the blood samples of individuals with CE. A key feature of this study was the comprehensive evaluation of the HAT performance using ROC curve analysis. The test revealed an AUC of 0.972, indicating an exceptionally high overall diagnostic accuracy. The optimal cutoff point (0.09375) achieved high sensitivity and specificity, each exceeding 95%, representing the most balanced trade-off between these parameters, as indicated by a Youden index of 0.903. Different cutoff values in the ROC analysis resulted in varying balances between sensitivity and specificity. A lower cutoff point contributes to increased sensitivity, making the assay more suitable for screening purposes, where minimizing false negatives is critical [58]. Conversely, a higher cutoff point enhances specificity, preferable for confirmatory testing where reducing false positives is essential. In this study, the selected optimal cutoff provided a balance appropriate for screening and confirmatory applications; however, specific adjustment of the cutoff value may be warranted depending on the intended diagnostic context [59].

The findings of our study indicated that the HAT test had a sensitivity of 95% and a specificity of 95.3%, determined by the cutoff point of 0.09375. Compared with previous studies, the results of the present study showed that the approaches used in test development increased its performance [38, 40], making it competitive with advanced instrument-based methods such as ELISA and western blot. Compared with this study, Erganis et al. [19] reported lower sensitivity and specificity for hemagglutination tests (66.67% and 92.31%, respectively). This difference is due to the hemagglutination method and kit used.

Some researchers modified the kit, improving its performance. By adding bacterial proteins (*Staphylococcus aureus* protein A), Parija et al. [30] improved sensitivity from 80.6% to 93.5%. In contrast, the present study achieved a sensitivity of 95% alone, without the need for additional reagents.

Recombinant antigens have also been used to increase the efficiency of serological testing using the ELISA method. Bashiri et al. increased the sensitivity and specificity of the test to 95% and 97.5%, respectively, using recombinant antigens [60]. According to the study by Masih et al., the sensitivity and specificity of the improved ELISA test with recombinant proteins were 92% and 95%, respectively [61]. At the same time, some studies have shown the sensitivity and specificity of the ELISA test to be over 95% [17, 53], consistent with the performance observed in the present study.

The methods of designing HAT in our study are similar to the study of Townsend et al. [27], which utilized a direct hemagglutination test to detect SARS-CoV-2 antibodies, reporting 90% sensitivity and 99% specificity. These findings confirm the potential applicability of the hemagglutination method across different diagnostic fields. Moreover, the sensitivity and specificity observed in this study were higher than those reported by previous commercial and laboratory-based hemagglutination kits [62, 63], emphasizing the importance of targeted refinement and redesign. The high sensitivity and specificity of the HAT and its low cost and operational simplicity make it an attractive tool for infectious disease diagnosis, particularly in resource-limited areas. These findings may contribute to the development of innovative diagnostic tests and the improvement of existing assays for both clinical and research purposes.

The determination of antibody titers using the HAT offers valuable clinical insights beyond simple positive and negative results. Elevated antibody titers are associated with active and severe CE infections [64, 65]. Moreover, longitudinal monitoring of antibody titers can help to assess treatment efficacy. The decreased levels of antibodies upon surgical and pharmacological intervention typically reflect successful treatment, whereas persistent and/or increased antibody levels may indicate incomplete removal, recurrence, and current infections [66]. Consequently, antibody titer assessment is crucial in the initial diagnosis, post-treatment monitoring, and long-term surveillance of CE [64].

This study has limitations, including a direct comparative assessment between ELISA and HAT. The IH4-fused antigen was selected owing to its high sequence conservation in *E. granulosus* sensu lato to enhance diagnostic sensitivity. Although preliminary in silico analysis has indicated minimal cross-reactivity with other *Taeniid* species, further experimental validation is necessary to confirm this finding and to explore the antigen’s potential applicability in veterinary diagnostics. We suggested more studies assessing cross-reactivity in clinical fields compared with standard techniques.

## Conclusions

This study presented a preliminary evaluation of a novel HAT for detecting anti-hydatid cyst antibodies using a recombinant fusion protein. The assay demonstrated acceptable sensitivity and specificity. However, comprehensive validation—including testing with sera from individuals infected with other parasitic diseases and further optimization—is required to clarify its diagnostic performance. Furthermore, more studies are needed to confirm this kit’s performance as a CE diagnostic tool.

## Supplementary Information

Additional file 1.

## Data Availability

All data supporting the findings of this study are included in the manuscript and its supplementary information file.

## Abbreviations

HAT: Hemagglutination test
RBCs: Red blood cells
ROC: Receiver operating characteristic
CE: Cystic echinococcosis
Ab: Antibody
Ag: Antigen
IHA: Indirect hemagglutination
RDTs: Rapid diagnostic tests
US: Ultrasonography
CT: Computed tomography
MRI: Magnetic resonance Imaging
SDS-PAGE: Sodium dodecyl sulfate polyacrylamide gel electrophoresis
PVDF: Polyvinylidene fluoride (membrane)
PBST: Phosphate-buffered saline with Tween 20
DAB: 3,3ʹ-Diaminobenzidine
BSA: Bovine serum albumin
IPTG: Isopropyl β-d-1-thiogalactopyranoside
AUC: Area under the curve
PPV: Positive predictive value
NPV: Negative predictive value
LFA: Lateral flow assay
OD: Optical density
TMB: Tetramethylbenzidine
