# Supplementary material for: Design and evaluation of a novel direct hemagglutination test based on a recombinant protein for diagnosis of cystic echinococcosis
Source: Parasit Vectors. 2025 Jul 8;18:267. doi: 10.1186/s13071-025-06900-1 (PMC12235937; doi:10.1186/s13071-025-06900-1)
Supplement: Supplementary file 1 — Additional file 1. [file 13071_2025_6900_MOESM1_ESM.docx]

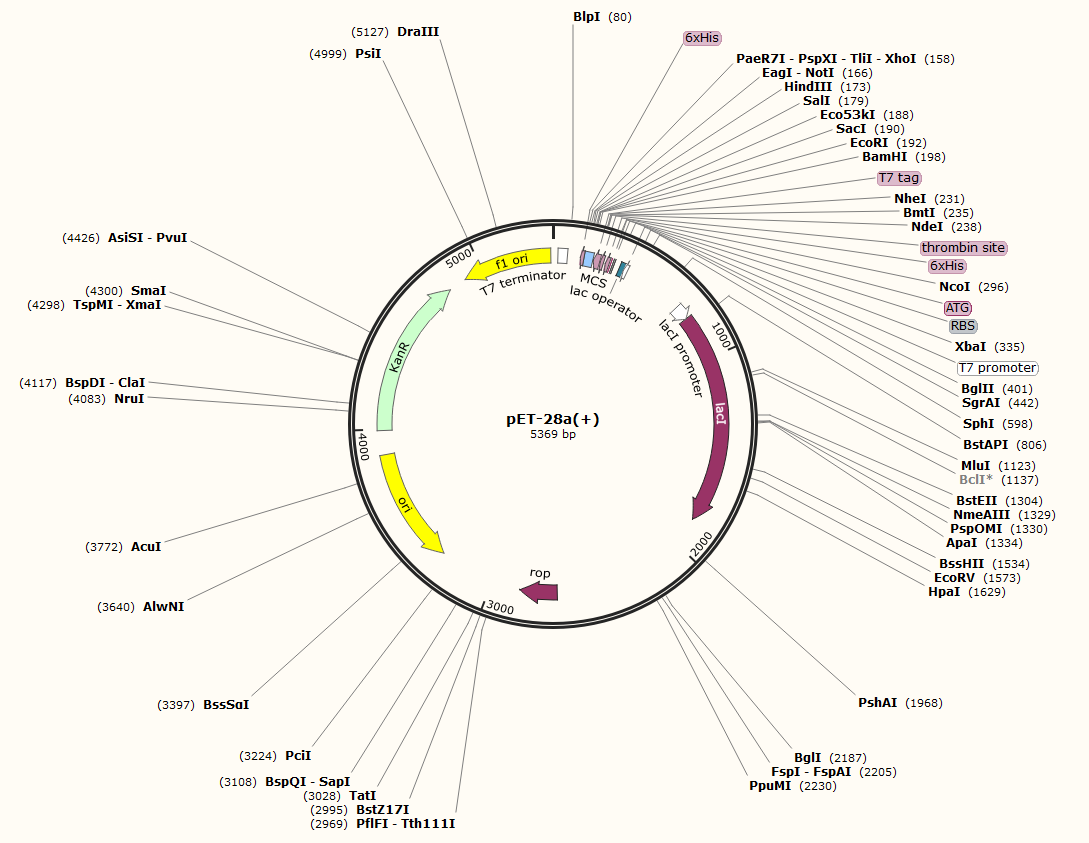


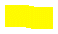

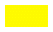


**Supplementary Figure S1: Plasmid map of IH4_Antigen_HisTag construct.**

**Supplementary Table S1: Amino acid sequence of the recombinant IH4_Antigen_HisTag protein.**

| MVHSQVQLQESGGGSVQAGGSLRLSCVASGYTDSTYCVGWFRQAPGKEREGVARINTISGRPWYADSVKGRFTISQDNSKNTVFLQMNSLKPEDTAIYYCTLTTANSRGFCSGGYNYKGQGTQVTVSSGSGSGTTMSRSEVGSGSGKSDMPTEMKGSGSGDTLREYNADSSRPIGSGSGLGRTLKQRYEGGSGSGKMEKSEGFGSGSGLGGGKYKMRSESTFKGSGSGKFKEVTPDSREVGSGSGKHEQDDKTKVGSGSGHHHHHH |
| --- |
| IH4  LINKER  ANTIGEN  His |


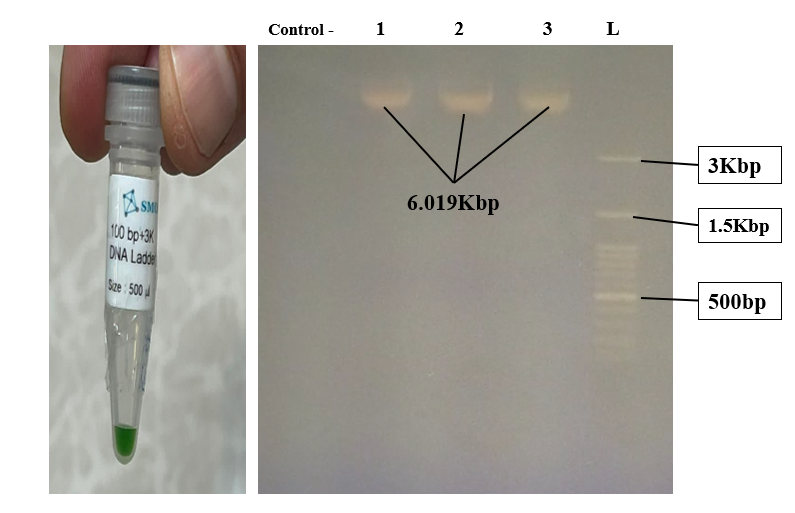


**Supplementary Figure S2: Agarose gel 1% electrophoresis analysis of extracted plasmids.**

All three plasmid samples exhibited a clear and sharp band of approximately 6 kb on the agarose gel. This observation indicates that the extracted plasmids contain the original pET28-a backbone (5369 bp) along with the cloned gene insert (788 bp) and linker sequences (138 bp), resulting in a total size of approximately 6019 bp, consistent with the expected construct size. No bands were observed in the negative control (BL21(DE3) cells without plasmid), confirming the absence of plasmid contamination or other unwanted DNA. The successful plasmid extraction and the sharpness of the bands reflect good sample purity and accuracy of the extraction process.

**Supplementary Table S2: Clinical Details of Positive Serum Samples**

| **Sample Code** | **Center** | **Cyst Location** | **Sample Volume (μl)** | **Diagnostic Method** | **Number** |
| --- | --- | --- | --- | --- | --- |
| 4026119310 | **hydatid.kmu^1^** | Liver | 200 | Imaging | 1 |
| 4026119342 | **hydatid.kmu** | Spleen | 200 | Imaging | 2 |
| 4026112662 | **hydatid.kmu** | Lung | 200 | Imaging | 3 |
| 4026112707 | **hydatid.kmu** | Lung | 200 | Imaging | 4 |
| 4026119329 | **hydatid.kmu** | Liver | 200 | Imaging | 5 |
| 4026119364 | **hydatid.kmu** | Lung | 100 | Imaging | 6 |
| 4026119355 | **hydatid.kmu** | Liver | 100 | Imaging | 7 |
| 4026113109 | **hydatid.kmu** | Lung | 100 | Imaging | 8 |
| 4026112686 | **hydatid.kmu** | Lung | 200 | Imaging | 9 |
| 4026119371 | **hydatid.kmu** | Liver | 200 | Imaging | 10 |
| 4026113130 | **hydatid.kmu** | Lung | 200 | Imaging | 11 |
| 4026113061 | **hydatid.kmu** | Lung | 100 | Imaging | 12 |
| 4026112612 | **hydatid.kmu** | Lung | 200 | Imaging | 13 |
| 4026113063 | **hydatid.kmu** | Lung | 200 | Imaging | 14 |
| 4026112591 | **hydatid.kmu** | Lung | 100 | Imaging | 15 |
| 4026113075 | **hydatid.kmu** | Lung | 200 | Imaging | 16 |
| 4026113053 | **hydatid.kmu** | Liver | 100 | Imaging | 17 |
| 4026112575 | **hydatid.kmu** | Liver | 200 | Imaging | 18 |
| 4026112800 | **hydatid.kmu** | Lung | 200 | Imaging | 19 |
| 4026117579 | **hydatid.kmu** | Liver | 200 | Imaging | 20 |
| 4026112775 | **hydatid.kmu** | Liver | 100 | Imaging | 21 |
| 4026112819 | **hydatid.kmu** | Lung | 200 | Imaging | 22 |
| 4026113177 | **hydatid.kmu** | Liver | 200 | Imaging | 23 |
| 4026113254 | **hydatid.kmu** | Lung | 200 | Imaging | 24 |
| 4026113283 | **hydatid.kmu** | Liver | 200 | Imaging | 25 |
| 4026113283 | **hydatid.kmu** | Lung | 100 | Imaging | 26 |
| 4026118150 | **hydatid.kmu** | Lung | 100 | Imaging | 27 |
| 4026117617 | **hydatid.kmu** | Lung | 100 | Imaging | 28 |
| 4026117451 | **hydatid.kmu** | Lung | 200 | Imaging | 29 |
| 4026117590 | **hydatid.kmu** | Liver | 200 | Imaging | 30 |
| 4026117644 | **hydatid.kmu** | Liver | 100 | Imaging | 31 |
| 4026118164 | **hydatid.kmu** | Liver | 200 | Imaging | 32 |
| 4026118173 | **hydatid.kmu** | Breast | 100 | Imaging | 33 |
| 4026118167 | **hydatid.kmu** | Lung | 100 | Imaging | 34 |
| 4026118152 | **hydatid.kmu** | Lung | 100 | Imaging | 35 |
| 4026118154 | **hydatid.kmu** | Lung | 200 | Imaging | 36 |
| 4026118184 | **hydatid.kmu** | Lung | 100 | Imaging | 37 |
| 4026117565 | **hydatid.kmu** | Liver | 100 | Imaging | 38 |
| 4026118171 | **hydatid.kmu** | Liver | 200 | Imaging | 39 |
| 4026117437 | **hydatid.kmu** | Liver | 200 | Imaging | 40 |
| - | **KAUMS^2^** | Liver | 500 | Imaging | 1 |
| - | **KAUMS** | Liver | 1000 | Imaging | 2 |
| - | **KAUMS** | - | 400 | ELISA | 3 |

1. Research Center for Hydatid Disease in Iran - Kerman University of Medical Sciences
2. Kashan University of Medical Sciences


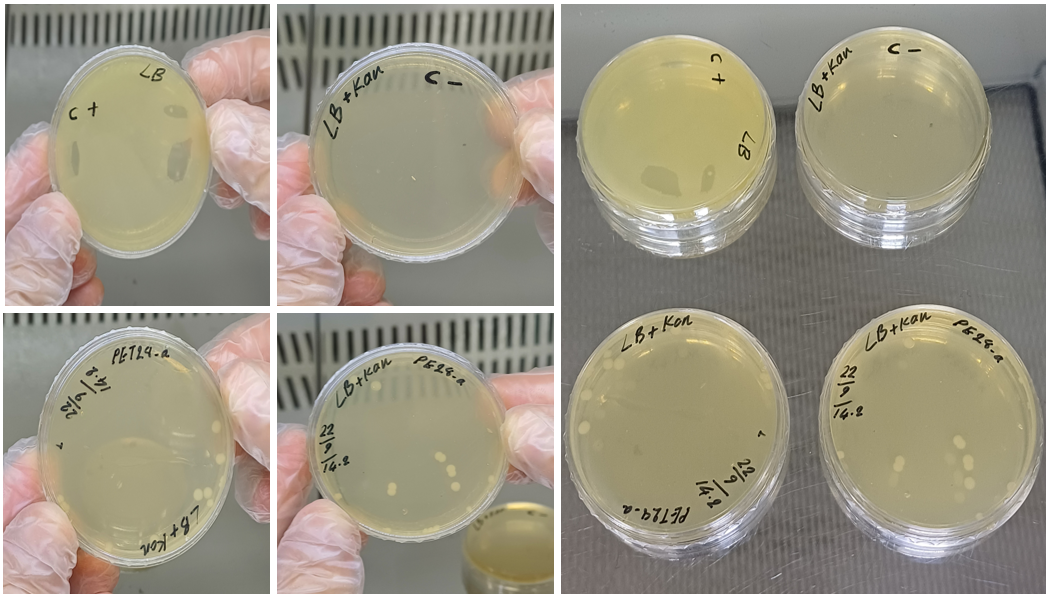


**Supplementary Figure S3:** Results and screening of transformed colonies along with negative and positive control. Positive control: bacteria without gene on LB medium without kanamycin, negative control: bacteria without gene on LB medium with kanamycin, two other culture mediums of transformed samples on LB medium with kanamycin.


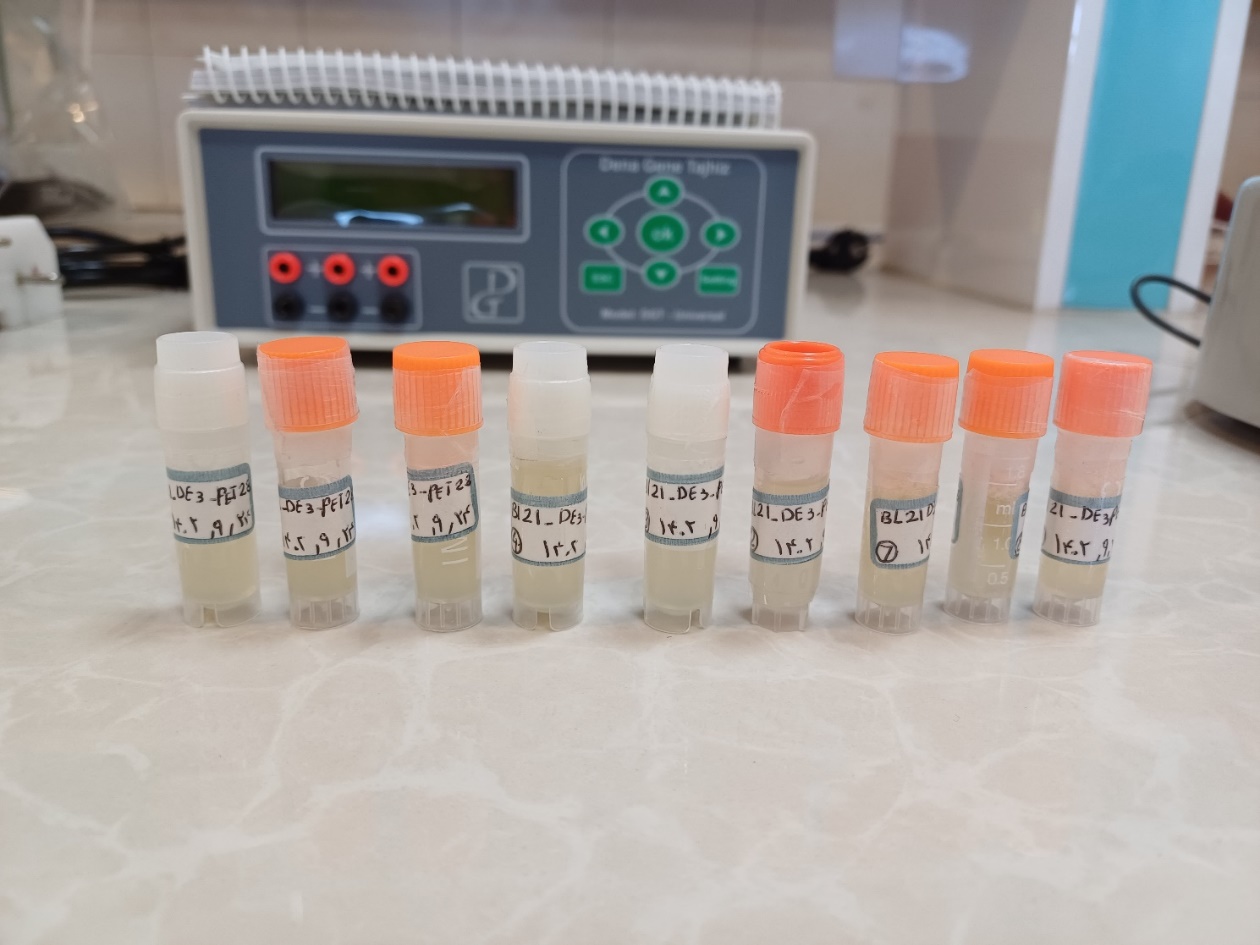


**Supplementary Figure S4:** Representative images of E. coli BL21(DE3) colonies harboring the IH4_Antigen_HisTag plasmid, grown on LB agar plates containing 50 μg/mL kanamycin. Glycerol stocks of selected colonies were prepared using 20% glycerol in LB broth and stored at 80°C for long term preservation.

**Sensitivity and Specificity Testing (raw data)**

Out of the 43 serum samples from patients with confirmed cystic echinococcosis (CE), 41 tested positive and 2 tested negative by the hemagglutination test. Among the 43 negative control samples, 41 tested negative and 2 tested positive. Based on these raw data, the preliminary diagnostic metrics were calculated as follows:

• **Sensitivity =** (41 / (41 + 2)) × 100 ≈ **95.34%**

• **Specificity =** (41 / (41 + 2)) × 100 ≈ **95.34%**
